# Role of Biologic Therapies in the Rheumatic Manifestations of Inflammatory Bowel Disease: A Systematic Analysis

**DOI:** 10.7759/cureus.45195

**Published:** 2023-09-13

**Authors:** Aiswarya Nag, Mansi Singh, Jingle Thomas, Rakshana Ravichandran, Lovish Gupta, Binay K Panjiyar

**Affiliations:** 1 Internal Medicine, Sri Ramachandra Institute of Higher Education and Research, Chennai, IND; 2 Department of Medicine, O.O. Bogomolets National Medical University, Kyiv, UKR; 3 Internal Medicine, Al-Ameen Medical College, Vijayapura, IND; 4 Internal Medicine, Rajarajeswari Medical College and Hospital, Bangalore, IND; 5 Internal Medicine, Maulana Azad Medical College, New Delhi, IND; 6 Department of Internal Medicine, Harvard Medical School, Boston, USA; 7 Internal Medicine, California Institute of Behavioral Neurosciences & Psychology, Fairfield, USA

**Keywords:** extraintestinal manifestations, spondyloarthropathies, peripheral arthritis, rheumatic manifestations, dual biologics, biosimilars, biologic therapy, ulcerative colitis, crohn’s disease, inflammatory bowel disease

## Abstract

In the recent years, there has been a growing recognition of the intricate relationship between inflammatory bowel disease (IBD) and extraintestinal manifestations (EIMs). EIMs of IBD include rheumatological, mucocutaneous, ocular, neurologic, pulmonary, cardiac, renal, hepatobiliary, and hematologic manifestations. Rheumatic manifestations are identified as the most common EIM, including axial and peripheral spondyloarthritis, arthralgia, sacroiliitis, enthesitis, and dactylitis. The convergence of the two distinct yet interconnected medical domains has spurred extensive research into the potential benefits of biological therapies as a treatment approach compared to the traditional method of treatment. This systematic review aims to assess the efficacy and overall impact of biological therapies in managing the rheumatic manifestations associated with IBD. Seventy-five articles from reputed journals published between January 1, 2013 and August 19, 2023 were reviewed. A set of eight papers were chosen for the focused study. The evaluation considered variables, such as rheumatic symptoms, in established IBD patients and compared the available biologic treatment and its benefits in alleviating rheumatic manifestations of IBD. By delving into the available literature and critically evaluating the relevant studies, this review shows insights into the role of biological therapies in the management of rheumatic symptoms in IBD. However, we must also address the limitations in implementing these since newer therapies are on the horizon. Hence, in-depth exploration and refinement of therapeutic strategies are needed to ultimately enhance patient care and quality of life for those affected by IBD. Infact, emerging artificial intelligence (AI) technologies are being used to improve the precision of diagnosis and enhance patient management.

## Introduction and background

Inflammatory bowel disease (IBD) is a chronic inflammatory condition affecting multiple organ systems, primarily the gut. Crohn's disease (CD) and ulcerative colitis (UC) have been categorized under IBD, which can present with extraintestinal manifestations (EIMs). Patients with CD tend to experience EIMs more frequently than individuals with UC [1]. The European Crohn’s and Colitis Organization (ECCO) classified these EIMs as musculoskeletal, mucocutaneous, ocular, neurologic, cardiac, pulmonary, renal, hepatobiliary, and hematologic, which is prevalent in around 6% to 46% of patients with IBD [2,3]. Chronic inflammation can eventually lead to colon cancer, widely recognized as a long-term complication of IBD [4]. Rheumatic manifestations are the most identified complications affecting 20% to 40% of patients with IBD [5-8]. The presentation can be extremely heterogeneous, ranging from articular to periarticular or even involving the bones and muscles [3]. Certain researchers have suggested that joint abnormalities could serve as the initial manifestation of IBD, and over time, individuals might subsequently develop pronounced intestinal abnormalities [9]. IBD-associated arthritis occurs in both males and females. Joint manifestations include both axial and peripheral arthropathies, arthralgia, sacroiliitis, enthesitis, and dactylitis [5]. Arthritis associated with IBD is a component of a subset of conditions broadly termed "seronegative spondyloarthropathies (SpA).” This group includes not only IBD-related arthritis but also conditions, such as psoriatic arthritis, reactive arthritis, and idiopathic ankylosing spondylitis (AS) [7]. Many patients with IBD are under multidrug treatment, and it is not always clear to which specific agent toxicity can be attributed. In addition, the underlying condition(s) can contribute to extraintestinal problems [10].

Peripheral arthritis is observed in 5% to 20% of individuals with IBD and tends to exhibit a recurrent pattern [3]. There are two types of peripheral arthritis: Type 1 peripheral arthritis is characterized by an asymmetric oligoarthritis affecting large joints, such as ankles, knees, hips, wrists, elbows, and shoulders, and is more prevalent and typically linked to IBD flares. By contrast, type 2 peripheral arthritis, which manifests as a symmetrical polyarthritis, affects small joints with a progressive nature and is unrelated to gut inflammation. The latter type exhibits greater aggressiveness and has the potential to lead to erosions [5,7]. The first type of arthritis is linked with disease activity, and consequently, addressing the underlying IBD is the preferred course of treatment. Conversely, the second type of IBD-associated arthritis often necessitates prolonged therapeutic intervention [3]. Colectomy may provide some protection against the development of type I peripheral arthritis, but it does not exert a notable impact on the trajectory of axial disease. Surgical procedures involving the small intestine typically do not serve as preventive measures against the onset of peripheral arthritis [11]. The connection between inflammation in the gut and joints in IBD is not yet entirely comprehended, although it has been researched extensively. Theories are attempting to explain the emergence of arthritis within the context of IBD. One revolves around gut bacteria, while the other focuses on the migration of gut lymphocytes to the joints. However, neither of these theories has been comprehensively elaborated upon. The prevalence of rheumatic manifestations reported exhibits considerable variability across various studies. These disparities could arise from differences in study methodologies for patient inclusion or diagnostic criteria applied to assess arthropathy [12].

The clinical characteristics of axial involvement encompass inflammatory back pain, AS, and sacroiliitis, each following an independent clinical trajectory [3]. The distinguishing features of axial spondyloarthritis are inflammation and bony proliferative changes, which include the development of syndesmophytes within the spine or the fusion of the sacroiliac joints [12]. Axial symptoms generally precede gut symptoms; the clinical trajectory remains entirely independent from intestinal manifestations and intestinal surgery does not influence the course of SpA. AS linked to IBD can manifest at any age, whereas idiopathic AS typically initiates before the age of 40 [9]. Clubbing, periostitis, and granulomatous diseases of the bones and joints are the other rheumatic manifestations [11]. Enthesitis involves inflammation at the insertion of a tendon to a bone, often observed at sites like the Achilles tendon or plantar fascia at the calcaneus. Conversely, dactylitis entails prolonged inflammation affecting the entire finger or toe, leading to a distinctive swollen appearance resembling a sausage. Chronic back pain is a vague symptom attributed to various origins, a higher prevalence of which was seen among individuals with IBD compared to what is expected within the general population [5]. Another articular EIM that can be attributed to either gut inflammation or treatment is septic arthritis. This condition can arise due to immunosuppressive therapy or be triggered by gut fistulas, potentially leading to bacterial infection in the iliosacral joint [3]. These manifestations, which often coexist with IBD, significantly impact patients' quality of life.

Diagnosis primarily relies on clinical assessment, based on the existence of peripheral or axial arthritis within the context of IBD [12]. The common laboratory findings include anaemia due to chronic inflammation, intestinal bleeding, leukocytosis, thrombocytosis, elevated acute phase reactants like C-reactive protein (CRP), and elevated erythrocyte sedimentation rate (ESR) [9]. However, key indicators of acute phase reaction, such as ESR and CRP, might be elevated in cases of active IBD, regardless of the presence of arthritis [11]. Antinuclear antibodies and rheumatoid factor are typically absent. Despite being an inflammatory condition, the synovial fluid is usually sterile [9]. Imaging is typically unnecessary for diagnosing IBD-associated peripheral arthritis; however, it might prove beneficial when evaluating other conditions. Plain radiographs of peripheral joints could reveal effusions or periarticular osteopenia similar to other inflammatory arthritides, but occurrences of erosions and joint deterioration are infrequent. Meanwhile, diagnosing axial arthritis often necessitates imaging procedures. MRI can contribute to the early identification of inflammatory conditions affecting the spine and sacroiliac region [12].

The principal objectives of medical treatment for IBD include initiating and maintaining clinical and endoscopic remission to treat symptoms, enhance the quality of life, and prevent complications that may result in hospitalization or surgical interventions [2]. In IBD patients with concurrent rheumatic involvement, biologics offer a comprehensive approach. For instance, some biologics can be used to treat both IBD and rheumatic manifestations simultaneously [4]. This integrated approach not only addresses gut inflammation but also alleviates joint pain and other musculoskeletal symptoms. Biological drugs, commonly known as biologics, have immensely evolved the management of IBD and its associated rheumatic manifestations. Traditional approaches involving physical therapy and corticosteroid injections have limitations and possibly harmful side effects on the integrity of the intestine, permeability, and even gut inflammation [9]. However, biologic therapies have shown promise in effectively addressing both intestinal inflammation and associated rheumatic symptoms [2]. These therapies target specific immune pathways, such as tumor necrosis factor (TNF), interleukins, and integrins, contributing to reduced inflammation in the gut and joints [4]. 

Among the most notable biologics used in IBD are anti-TNF agents, which were first introduced in the 1990s, such as infliximab and adalimumab [11]. These drugs target TNF-alpha, a pro-inflammatory cytokine implicated in both IBD and rheumatic conditions. By neutralizing TNF-alpha, anti-TNF biologics interrupt the inflammatory cascade at a crucial point. TNF inhibitors have demonstrated remarkable efficacy for IBD patients who rely on steroids or are unresponsive to conventional treatments [12]. A chimeric monoclonal antibody targeting TNF-alpha called infliximab, composed of 75% human and 25% murine sequences, has been extensively studied in the context of IBD [4]. In cases of moderate to severe CD and UC, infliximab is particularly potent, facilitating fistula closure, promoting mucosal healing, and reducing the need for steroid administration [12]. Adalimumab is a human immunoglobulin G1 (IgG1) monoclonal antibody designed to target TNF. In a clinical trial, adalimumab demonstrated superior efficacy compared to placebo in inducing remission among patients with moderate to severe CD who were previously untreated with anti-TNF therapy [11].

Interleukin-12 is the principal inducer of Th1 immune responses in CD and is primarily produced by monocytes/macrophages following bacterial stimulation. In addition, the innate immune system and the IL-23/T helper-17 (Th17) axis have been implicated as pivotal factors in the development of IBD. Ustekinumab (UST) is a human IgG1κ monoclonal antibody that blocks the biological functions of IL-12 and IL-23 cytokines by targeting their shared p40 subunit. These cytokines play a role in the underlying mechanisms of CD. IL-17 triggers the migration of immune cells to peripheral tissues, a process dependent on the activation of nuclear factor kappa B (NF-kB) following engagement with the IL-17 receptor. Moreover, IL-17 induces the generation of numerous pro-inflammatory agents, such as TNF-alpha, IL-6, IL-23, and IL-1b, by innate immune cells and antigen-presenting cells. Studies examining the effectiveness of UST in individuals with active AS were halted as the drug failed to attain significant outcomes [4]. Vedolizumab (VDZ) is a humanized monoclonal antibody that inhibits the entire α4β7 integrins within the gastrointestinal tract by inhibiting the interaction between α4β7 and mucosal addressin cell adhesion molecule 1 (MAdCAM-1). Unlike some treatments, VDZ does not usually lead to systemic effects, as it specifically disrupts lymphocyte migration solely within the gastrointestinal tract [5]. Tocilizumab, a humanized monoclonal antibody targeting the IL-6 receptor and currently employed in rheumatoid arthritis treatment, demonstrated a greater clinical response rate in comparison to a placebo. However, the treatment did not yield endoscopic or histological healing effects. Numerous humanized monoclonal antibodies that specifically target the p19 subunit of IL-23 are currently being explored for their potential in IBD treatments [4].

Janus kinases (JAKs) play a pivotal role in both innate and adaptive immune responses. Numerous cytokines implicated in IBD pathogenesis utilize the JAK/STAT pathway for signalling. Pro-inflammatory cytokines that utilize this pathway include interleukins, such as IL-2, IL-6, IL-12, IL-23, and IFN-gamma. Conversely, cytokines, such as TNF, IL-1, IL-8, TGF-β, and macrophage colony-stimulating factors, do not employ the JAK/STAT pathway for signaling. Tofacitinib is an orally administered pan JAK inhibitor that effectively targets JAK1, JAK2, and JAK3, as well as TYK2 to a lesser extent. Small molecules, such as tofacitinib, offer advantages compared to biologic agents, including reduced risk of immunogenicity and the convenience of oral administration [4]. UST has been extensively studied particularly following the ineffectiveness of conventional treatments and anti-TNF agents [13]. 

Biosimilars are biotherapeutic substances engineered to replicate molecules similar to those found in an established reference product, including factors, such as amino acid sequence, post-translational modifications, protein conformation, pharmacokinetics, receptor interaction, post-receptor impacts, immunogenicity, safety, and effectiveness. Biosimilars could offer cost-effective alternatives close to generic versions of medications [5]. Sequential and combination biologic therapies have been explored to enhance outcomes. While challenges, such as infection risk, exist, studies emphasize the benefits of biologic therapies in managing IBD and associated rheumatic manifestations, transforming patient care [8]. In the present scenario of IBD treatments, numerous biologic alternatives with comparable and substantial effectiveness on IBD activity are available. Hence, selecting the most suitable biologic for a particular patient can be challenging, particularly when EIMs are present [2]. Biologics target specific immune pathways and molecules implicated in both IBD and musculoskeletal symptoms, and these drugs provide a comprehensive approach to disease management. While challenges exist, the benefits of biologics in transforming patient care and improving the quality of life are undeniable.

## Review

Methods

**Figure 1:**
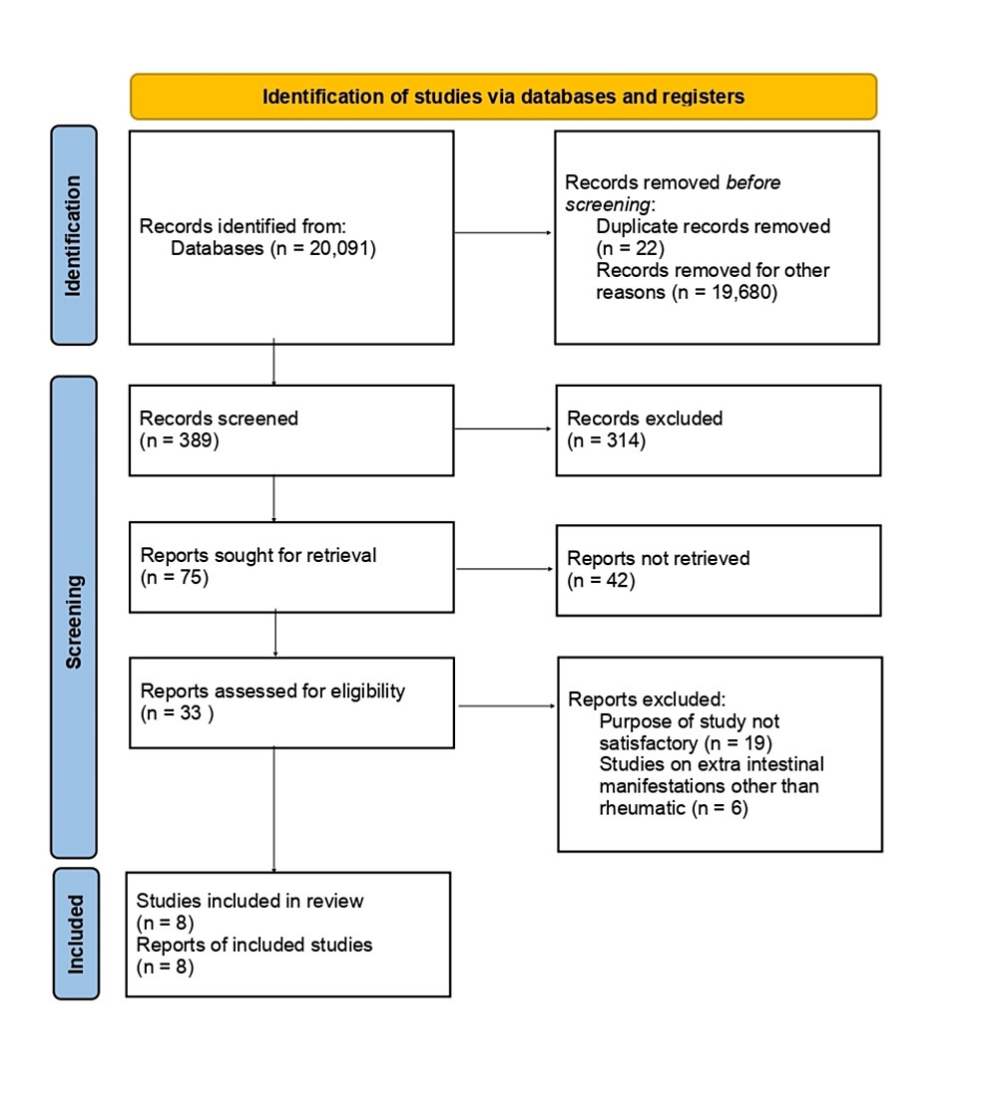
PRISMA flow diagram illustrating the search strategy and study selection process for the systematic review. PRISMA: Preferred Reporting Items for Systematic Reviews and Meta-Analyses

This review focuses on the systematic analysis of biological interventions' impact in treating the rheumatic symptoms of IBD. We omitted research involving animals and papers that did not discuss the extraintestinal involvement of IBD. The review follows the guidelines of the Preferred Reporting Items for Systematic Reviews and Meta-Analyses (PRISMA) [14] for 2020 in Figure 1 and only uses data collected from published papers, eliminating the need for ethical approval.

Systematic Literature Search and Study Selection

We extensively explored relevant literature through PubMed, encompassing MEDLINE and Google Scholar. Our investigation encompassed research indicated in review articles, editorials, and commentaries available on PubMed. Nonetheless, we persisted in seeking supplementary studies that met our established criteria.

We had a variety of abstracts that underwent individual assessment for eligibility according to defined criteria. These criteria encompassed the rheumatic manifestations of IBD, the impact of newer emerging biologic therapies in management, and a well-defined clinical cohort within the research. We omitted review articles and studies involving animals. A team of six reviewers (AN, MS, JT, RR, LG, and BP) independently conducted an evaluation, and disparities were resolved through discussion.

Inclusion and Exclusion Criteria

We established specific criteria for including and excluding participants to achieve our study goals. Our criteria can be summarized in Table 1. 

**Table 1:** Criteria used during the literature search process.

	Inclusion criteria	Exclusion criteria
1	Human studies	Animal studies
2	Articles from 2013 to 2023	Only methodological studies explaining programming details
3	Only English language	Non-English articles
4	Both male and female	
5	Age > 19 years	Age <= 19 years
6	Free full-text papers	Papers that needed a subscription
		Studies involving clinical data other than inflammatory bowel disease

Search Strategy

The population, intervention/condition, control/comparison, and outcome (PICO) criteria were used to conduct a thorough literature review. The search was conducted on databases, such as PubMed (including MEDLINE) and Google Scholar libraries, using relevant keywords, such as "inflammatory bowel disease," "rheumatic," "extraintestinal," and "biologic therapy." The medical subject heading (MeSH) approach for PubMed (including MEDLINE) and Google Scholar, as detailed in Table 2, was employed to develop a comprehensive search strategy. 

**Table 2:** Search strategy, search engines used, and the number of results displayed.

	Database	Search strategy	Search results
A	PubMed	(Inflammatory bowel disease OR Crohn’s disease OR Ulcerative colitis) AND ( rheumatic) AND (biologic therapy)	289
		(Inflammatory bowel disease) AND (biologic therapies)	2
B	Google Scholar	(Inflammatory bowel disease OR Crohn's disease OR Ulcerative colitis) AND (rheumatic manifestations OR extra intestinal) AND (biological therapies)	19,800

Quality Appraisal

To ascertain the credibility of our selected papers, we employed diverse tools for evaluating their quality. The PRISMA checklist and Cochrane risk-of-bias assessment tool for randomized clinical trials were applied to systematic reviews and meta-analyses. Non-randomized clinical trials underwent assessment using the Newcastle-Ottawa scale. The quality assessment of qualitative studies, as presented in Table 3, involved the Critical Appraisal Skills Program (CASP) checklist. To prevent ambiguity in classification, we employed the Scale for the Assessment of Narrative Review Articles (SANRA) for evaluation.

**Table 3:** Quality appraisal tools used. RCT: randomized controlled trials; PRISMA: Preferred Reporting Items for Systematic Reviews and Meta-Analyses; SANRA: Scale for the Assessment of Narrative Review Articles

Quality appraisal tools used	Type of studies
Cochrane risk-of-bias assessment tool	Randomized control trials (RCTs)
Newcastle-Ottawa tool	Non-RCT and observational studies
PRISMA checklist	Systematic reviews
SANRA checklist	Any other without a clear method section

Results

Following an extensive exploration across the three chosen databases, namely, PubMed, MEDLINE, and Google Scholar, we accumulated a total of 20,091 articles. Subsequently, we meticulously assessed each paper against specific criteria, leading to the exclusion of 19,680 articles. Among the remaining 411 papers, we opted not to incorporate 336 due to duplication or unsatisfactory titles and abstracts. We focused on the remaining 75 papers, and 67 more were ruled out due to their content not matching our inclusion parameters. Finally, a comprehensive quality assessment was conducted on the remaining eight papers, all of which satisfied our criteria. These eight articles constitute the core of our ultimate systematic review. A comprehensive breakdown of each paper is presented in Table 4.

**Table 4:** Summary of the results of the selected papers. UST: Ustekinumab; UNITI: Unification of Treatments and Interventions for Tinnitus patients; IBD: inflammatory bowel disease; EIM: extraintestinal manifestation; TNF-α: tumor necrosis factor-α

Author/Year	Country	Study design	Databases used	Conclusion
Narula et al./2021 [1]	Canada, Saudi Arabia, Austria	Post-hoc analysis	PubMed, Google Scholar, Web of Science	Ustekinumab did not resolve extraintestinal manifestations of IBD in comparison to placebo.
Ferreti et al./2022 [2]	Italy	Retrospective cohort study	PubMed, Google Scholar, Crossref	The choice of biologic treatment might influenced the development of new-onset EIMs, particularly those on gut-sensitive therapies.
Colin et al./2016 [3]	Italy	Comprehensive review	PubMed, Google Scholar, Web of Science	IBD presents as a complex inflammatory condition with possible multiple organ involvement.
Park/2020 [4]	Korea	Systematic literature review	PubMed, Crossref	Collaborative communication between gastroenterologists and rheumatologists may be necessary since drugs used in rheumatic symptoms may deteriorate IBD.
Sheth et al./2015 [5]	USA	Systematic literature review	PubMed, Google Scholar, Crossref	Anti-TNF-α agents have revolutionized the joint manifestations of IBD with newer biologics and biosimilars emerging on the horizon.
Guillo et al./2020 [6]	France, Italy	Systematic review	PubMed, Embase, Web of Science	Biological drugs resolve EIMs of IBD with varying degrees; however, a collaborative approach of dedicated specialists is required to ensure the best patient care.
Privitera et al./2021 [8]	Italy	Review article	PubMed, Google Scholar, Crossref	The review explored the potential role of biologics and a more dynamic approach to IBD treatment, involving sequencing and combining targeted therapies.
Berg et al./2019 [13]	USA	Systematic literature review	PubMed, Google Scholar, Crossref, WorldCat	The treatment approach for IBD has been evolving from a traditional "step-up" strategy, starting with less potent therapies and escalating treatment as needed to a "top-down" early intervention approach using biologics.

Discussion

Conventional treatments for IBD comprise 5-aminosalicylic acid(5-ASA)/aminosalicylates/mesalamine, steroids, and immunomodulators. Immunomodulators, such as azathioprine, mercaptopurine, and methotrexate, serve as valuable supplementary treatments, offering safer and better tolerated options compared to prolonged steroid therapy. However, the landscape has transformed with the advent of anti-TNF agents, which have significantly altered the management approach in recent times [15]. IBD-associated SpA often receives physical therapy and non-steroidal anti-inflammatory drugs (NSAIDs) to alleviate pain, inflammation, and rigidity. While rheumatologists frequently employ NSAIDs with positive outcomes, their use remains controversial among IBD patients [16]. The majority of patients typically exhibit a positive response to NSAIDs due to their ability to manage symptoms and mitigate joint and enthesis inflammation [9]. It is commonly believed that COX-2 inhibitors have a lower chance of inducing intestinal flares compared to COX-1 inhibitors [17]. However, NSAIDs can potentially cause gut toxicity and hence should be avoided. While NSAID-related enteropathy might not present symptoms in some cases, certain individuals could encounter small gut bleeding and protein loss. The occurrence of significant gut ulcers is infrequent. Notably, NSAID administration can trigger IBD exacerbations, particularly in individuals with CD. While 5-ASA is widely regarded as the benchmark treatment for sustaining IBD remission, it does not offer significant benefits for addressing joint complications [11].

In a population-based prospective cohort by Ossum et al., 470 people were investigated for IBD over a 20-year observation period. SpA was reported with an overall prevalence ranging from 17% to 39%. On the contrary, Vavricka et al. discovered no correlation between the activity of IBD (as measured by the Crohn’s Disease Activity Index (CDAI) for CD and the Truelowe-Witts severity index for UC) and the occurrence of AS. Human leukocyte antigen (HLA-B27) displayed associations with AS, axial SpA, and inflammatory back pain, while neucleotide-binding oligomerization domain 2 (NOD2) exhibited no substantial correlation. The study also confirmed that HLA-B27 puts both AS and axial SpA at risk. Furthermore, a more chronic course of IBD was associated with axial SpA [18]. HLA-B27's role in the development of arthritis in IBD involves presenting arthritogenic peptides to T cells, which can trigger inflammation. In addition, this genotype might predispose to protein misfolding, further contributing to inflammation. However, it is important to note that while HLA-B27 plays a part, it only accounts for a fraction of the overall genetic susceptibility to arthritis in IBD. An IL-23R polymorphism that has been linked to a reduced risk of developing IBD has also been observed to confer protection against AS. This suggests a potential shared genetic mechanism that influences susceptibility to both conditions [12].

Exciting therapeutic prospects have emerged with the recent introduction of biologic agents, notably anti-TNF agents. Infliximab, which neutralizes both soluble and membrane-bound cytokines, shows promise for UC. Its efficacy in treating fistulas and severe CD is well established. Moreover, a notable improvement in joint-related and axial symptoms has been observed in a limited cohort of CD patients. Infliximab's effectiveness in treating AS and psoriatic arthritis is widely recognized [11]. Orlando et al. demonstrated the potential benefit of vedolizumab (VDZ) on IBD-associated SpA. A proportion (46.2%) of patients with active SpA had clinical improvement with the induction of VDZ, and no flare of arthritis/sacroiliitis was reported [19]. Natalizumab was found effective in both luminal symptoms and polyarthritis [20]. A randomized placebo-controlled trial has also suggested the effectiveness of adalimumab in treating AS. Researchers have illustrated rapid enhancement in peripheral arthritis among IBD patients undergoing Infliximab treatment [21]. An open pilot study reported the resolution of arthralgia in 63.6% of IBD patients following a single infusion of infliximab. Apart from inducing remission in individuals with AS, infliximab has demonstrated effectiveness in sustaining remission as well [7]. However, in a post-hoc analysis by Narula et al., treatment of arthritis/arthralgia associated with IBD with ustekinumab (UST) showed no significant resolution compared to placebo at weeks 6 and 52. The insignificant findings could potentially be due to the differential use of concomitant therapies, considering that the use of systemic steroids and immunomodulators was slightly more prevalent among those receiving the placebo [1]. Investigations are being conducted into anti-trafficking therapies sphingosine-1-phosphate receptor modulators, such as ozanimod and etrasimod, in UC. Sphingosine-1-phosphate (S1P) is a signalling molecule that controls the movement of lymphocytes from lymphoid organs into the bloodstream and inflamed tissues. These therapies aim to manipulate this signaling pathway for potential therapeutic benefits in UC [4].

VDZ and UST have shown promising results in patients with EIMs, particularly for those with articular complaints. However, they may not be as effective for managing axial spondylarthritis [22]. The total incidence of new-onset EIMs in patients undergoing VDZ treatment was statistically greater in comparison to those receiving non-gut selective therapies according to a study by Ferreti et al. Older age, female gender with UC, an extended duration of biologic therapy, and the use of gut-selective agents were associated with a higher risk of new-onset EIMs [2]. Patients administered with VDZ showed a higher incidence of deterioration in pre-existing rheumatic EIMs, although this trend did not attain statistical significance. These findings align with Ramos et al.'s observations, where nearly a third of 201 patients receiving VDZ experienced a deterioration in preexisting EIMs, primarily affecting peripheral arthritis [2]. Keeping aside the possibility of new onset or exacerbation of arthritis/sacroiliitis on induction of VDZ, recent evidence of increased expression of mucosal vascular addressin cell adhesion molecule (MadCAM-1) in the high endothelial venules of bone marrow among individuals with active axial SpA appears to reinforce the notion of a favorable rather than counterintuitive effect of α4β7 blockade on the joint-related manifestations of IBD [19]. Therefore, for patients with concomitant risk factors for EIMs, it is important to contemplate therapeutic approaches beyond VDZ as the primary choice, if feasible. Alternatively, when opting for VDZ treatment, vigilant monitoring for the emergence of rheumatic symptoms is recommended [2]. In a study by Momen Majumder et al., tofacitinib, which is a pan JAK inhibitor administered orally, is suggested as an effective treatment option for IBD-associated arthritis, particularly when conventional disease-modifying antirheumatic drugs (DMARDs) have failed. It may also be beneficial when biologic treatments prove ineffective for managing IBD-associated arthritis. However, its ability to sustain remission over an extended period is still under investigation in longer-term studies [23,24].

TNF-α plays a crucial role in the immune response, particularly during tuberculosis (TB) infection. It activates macrophages, recruits immune cells, supports granuloma formation, and maintains granuloma stability [15]. Hence, an important aspect in the inhibition of TNF-α is the reactivation of latent TB or an increased susceptibility to primary TB infection, a phenomenon that was not identified during the initial trials involving infliximab [10]. This was predominantly seen in Asia, where over 60% of the 10.4 million new TB cases in 2016 were documented. Numerous studies have highlighted the high risk of TB associated with anti-TNF agents, even in patients who initially tested negative for TB before commencing treatment. The clinical manifestations of TB infection in such cases might resemble those seen in immunocompromised individuals, including atypical, miliary, or extrapulmonary TB. This increased TB risk may arise due to its impact on cell-mediated immunity; anti-TNF agents could reduce the number of CD8+ cells responsible for countering *Mycobacterium tuberculosis*. Particularly, in TB-endemic countries, this risk of TB reactivation has significant implications for managing IBD. All IBD patients should undergo TB screening before commencing anti-TNF treatments. The challenge is exacerbated by the difficulty in distinguishing between CD and gastrointestinal TB during diagnosis. Making the correct diagnosis is crucial due to the serious implications of initiating the wrong treatment in cases of misdiagnosis. VDZ emerges as a promising option for IBD management, particularly in regions with a high prevalence of TB, such as the Asia-Pacific. Hence, VDZ could potentially serve as a primary choice for biologic treatment in IBD management, especially when TB is a critical consideration in clinical decision-making [15]. Moreover, anti-TNF therapy has been linked to the reactivation of hepatitis B infection [23].

Dual therapy for IBD was popularized in 2010 when a trial by the Study of Biologic and Immunomodulator Naive Patients in Crohn’s Disease (SONIC) was released. Findings from the SONIC trial indicated that individuals with mild to moderate CD who were treated with a combination of infliximab and azathioprine had a higher likelihood of attaining corticosteroid-free remission compared to those who received azathioprine alone. The use of dual biologic therapy has been suggested in the treatment strategy for two categories of patients with IBD: those with well-managed luminal IBD and uncontrolled extra-intestinal symptoms (e.g., arthritis or psoriasis) and those with persistent, unmanageable IBD [25]. Even within therapies that target TNF, varying responses are observed across different disease contexts. As a result, there is a clear need for enhanced methods to identify and predict which patients are most likely to exhibit positive responses to particular individual treatments or specific combinations of therapies [26]. Patients undergoing combination therapy involving infliximab and natalizumab displayed to have enhanced clinical outcomes. This was evident in the form of higher rates of clinical remission, in contrast to patients who received infliximab along with a placebo. Researchers have initiated assessments of the safety and effectiveness of combination therapy using more recent biologics, which have enhanced safety profiles [25]. Moreover, evidence indicates that incorporating immunomodulators could offer advantages to certain patients experiencing a decline in response to anti-TNFα therapy [8].

Infections and septicemia have unquestionably been the primary contributors to mortality in patients with IBD. Common infections associated with anti-TNF therapy are upper respiratory tract and urinary infections. The emergence of lymphoma has been a significant concern for both patients and doctors using biologic therapy, especially following reports in which patients develop non-Hodgkin’s lymphoma while undergoing treatment with infliximab and immunomodulators. Immunogenicity is a great concern linked to all monoclonal antibodies, including the humanized molecules. This antibody formation can be prevented through the systematic administration of biologics rather than on-demand therapy. Moreover, administering hydrocortisone before infusion can prevent immunogenicity [10]. Despite the increase in the relative risk of infections and neoplasms linked to the use of biologic agents, the actual risk remains very minimal, even when considering the potential interference of concurrent therapies [26]. Initiating intensive biologic therapy early in the disease course enables mucosal healing and has the potential to halt the advancement toward structural bowel damage [13]. The significance of intervening early in a progressive structural disease process highlights that the use of anti-TNF and other biologic treatments can prevent the progression of structural damage if administered before the onset of joint (or bowel wall) [26].

IBD remains highly heterogeneous in its course. Certain individuals sustain mild disease manifestations, while others undergo rapid disease progression. The primary aim of initiating biologic therapy early is to intervene in the disease's natural progression and prevent the emergence of future complications. Furthermore, IBD frequently impacts individuals during their most productive years, and achieving remission can reduce indirect costs linked to unemployment and absenteeism. Undoubtedly biologic therapy comes with a high cost and currently, it stands as a principal contributor to healthcare expenditures. The introduction of biosimilars into the pharmaceutical market is anticipated to contribute to cost reduction. With the gradual increase in the availability of biosimilars, competition is expected to cut down on the prices, ultimately leading to reduced expenses for patients and facilitating broader access to biologic therapy [13]. Biosimilar agents, owing to their intricate composition, might not be entirely identical to the reference product, although their active ingredients are fundamentally the same. The Food and Drug Administration (FDA) permits minor variations in clinically inactive components within biosimilar products [23]. The biosimilar CT-P13, a version of infliximab, was introduced in June 2013 and marked the debut of a biosimilar monoclonal antibody for IBD treatment [5]. Numerous studies have indicated that CT-P13 demonstrates similar effectiveness and safety in comparison to other treatments for inducing and maintaining remission in patients with IBD [23]. Biosimilars for adalimumab, rituximab, and etanercept are undergoing diverse phases of clinical trials and are anticipated to become available in the market within the next decade [5]. Eventually, this will lead to the earlier initiation of biologics in patients who might have otherwise delayed treatment until complications arose. Initiating such a therapy early is likely to result in diminished long-term treatment costs [13]. Artificial intelligence (AI) is demonstrating great promise in the field of clinical research related to IBD endoscopy. Emerging AI technologies have the potential to enhance the efficiency and accuracy of evaluating the initial endoscopic findings in IBD patients and assessing how various therapeutic interventions impact mucosal healing. These advancements in AI can significantly benefit both the diagnosis and treatment monitoring of IBD, improving patient care and outcomes [27].

Patients with IBD-associated arthropathy commonly present to gastroenterology first, which subsequently leads to a referral to rheumatology for in-depth assessment and care. A comprehensive articular examination, encompassing both axial and appendicular aspects, is essential. In very few cases, patients may initially approach rheumatology, and a thorough review of their medical history could unveil underlying gastrointestinal issues. In such scenarios, a prompt referral to gastroenterology for thorough evaluation is recommended, given that immunomodulatory therapy might obscure endoscopic findings and postpone the diagnosis of IBD [12]. Hence, a holistic approach involving gastroenterologists and rheumatologists might change the clinical course of patients and reduce mortality.

Limitations

Our examination of the literature has limitations. Our analysis was constrained to articles published within the last 10 years, with a specific focus on those aged at least 19 years. Moreover, our review was restricted to freely accessible articles and our search was confined to English-language papers that explored the rheumatic manifestations of IBD and how biologic therapy has an impact. Nonetheless, further research is imperative to draw precise conclusions.

## Conclusions

IBD and rheumatic manifestations often coexist, necessitating a holistic approach to treatments. Biologic therapies have emerged as a promising avenue in managing these complex medical scenarios. This systematic review underscores the consistent evidence supporting the efficacy of biologic agents in alleviating rheumatic symptoms in IBD patients. By providing an overview of existing research, identifying gaps in knowledge, and emphasizing the need for tailored treatment strategies, this review contributes to the advancement of patient care in this challenging clinical intersection. Further research and collaboration between gastroenterologists and rheumatologists hold the key to refining biologic interventions and enhancing the overall well-being of individuals with these overlapping conditions.
